# Comparison of index-linked HIV testing for children and adolescents in health facility and community settings in Zimbabwe: findings from the interventional B-GAP study

**DOI:** 10.1016/S2352-3018(20)30267-8

**Published:** 2020-11-13

**Authors:** Chido Dziva Chikwari, Victoria Simms, Katharina Kranzer, Stefanie Dringus, Rudo Chikodzore, Edwin Sibanda, Karen Webb, Barbara Engelsmann, Nicol Redzo, Tsitsi Bandason, Hilda Mujuru, Tsitsi Apollo, Getrude Ncube, Karen Hatzold, Helen A Weiss, Rashida A Ferrand

**Affiliations:** a Clinical Research Department, London School of Hygiene & Tropical Medicine, London, UK; b Medical Research Council Tropical Epidemiology Group, London School of Hygiene & Tropical Medicine, London, UK; c Biomedical Research and Training Institute, Harare, Zimbabwe; d Ministry of Health and Child Care, Bulawayo, Zimbabwe; e Health Services Department, Bulawayo, Zimbabwe; f Organization for Public Health Interventions and Development, Harare, Zimbabwe; g Department of Paediatrics, University of Zimbabwe, Harare, Zimbabwe; h AIDS and Tuberculosis Unit, Ministry of Health and Child Care, Harare, Zimbabwe; i Population Services International, Harare, Zimbabwe

## Abstract

**Background:**

Index-linked HIV testing, whereby children of individuals with HIV are targeted for testing, increases HIV yield but relies on uptake. Community-based testing might address barriers to testing access. In the Bridging the Gap in HIV testing and care for children in Zimbabwe (B-GAP) study, we investigated the uptake and yield of index-linked testing in children and the uptake of community-based *vs* facility-based HIV testing in Zimbabwe.

**Methods:**

B-GAP was an interventional study done in the city of Bulawayo and the province of Matabeleland South between Jan 29 and Dec 12, 2018. All HIV-positive attendees (index patients) at six urban and three rural primary health-care clinics were offered facility-based or community-based HIV testing for children (age 2–18 years) living in their households who had never been tested or had tested as HIV-negative more than 6 months ago. Community-based options involved testing in the home by either a trained lay worker with a blood-based rapid diagnostic test (used in facility-based testing), or by the child's caregiver with an oral HIV test. Among consenting individuals, the primary outcome was testing uptake in terms of the proportion of eligible children tested. Secondary outcomes were uptake of the different HIV testing methods, HIV yield (proportion of eligible children who tested positive), and HIV prevalence (proportion of HIV-positive children among those tested). Logistic regression adjusting for within-index clustering was used to investigate index patient and child characteristics associated with testing uptake, and the uptake of community-based versus facility-based testing.

**Findings:**

Overall, 2870 index patients were linked with 6062 eligible children (3115 [51·4%] girls [sex unknown in seven], median age 8 years [IQR 5–13]). Testing was accepted by index patients for 5326 (87·9%) children, and 3638 were tested with a known test outcome, giving an overall testing uptake among 6062 eligible children of 60·0%. 39 children tested positive for HIV, giving an HIV prevalence among the 3638 children of 1·1% and an HIV yield among 6062 eligible children of 0·6%. Uptake was positively associated with female sex in the index patient (adjusted odds ratio [aOR] 1·56 [95% CI 1·38–1·77], p<0·0001) and child (aOR 1·10 [1·03–1·19], p=0·0080), and negatively associated with any financial cost of travel to a clinic (aOR 0·86 [0·83–0·88], p<0·0001), increased child age (6–9 years: aOR 0·99 (0·89–1·09); 10–15 years: aOR 0·91 [0·83–1·00]; and 16–18 years: aOR 0·75 [0·66–0·85]; p=0·0001 *vs* 2–5 years), and unknown HIV status of the mother (aOR 0·81 [0·68–0·98], p=0·027 *vs* HIV-positive status). Additionally, children had increased odds of being tested if community-based testing was chosen over facility-based testing at screening (1320 [73·9%] children tested of 1787 *vs* 2318 [65·5%] of 3539; aOR 1·49 [1·22–1·81], p=0·0001).

**Interpretation:**

The HIV yield of index-linked testing was low compared with blanket testing approaches in similar settings. Index-linked HIV testing can improve testing uptake among children, although strategies that improve testing uptake in older children are needed. Community based testing by lay workers is a feasible strategy that can be used to improve uptake of HTS among children and adolescents.

**Funding:**

UK Medical Research Council, UK Department for International Development, Wellcome Trust.

## Introduction

The scale-up of antiretroviral therapy (ART) globally in the past 20 years has substantially reduced HIV-associated mortality across all age groups.^1^ However, globally the coverage of ART in children (<15 years) was only 53% in 2019, largely due to delays in diagnosis.^2^ Testing of HIV-exposed children within prevention of mother-to-child transmission (PMTCT) programmes remains suboptimal, and subsequently children with perinatally acquired HIV are often diagnosed later in childhood when they present with HIV-associated sequelae.^3^, ^4^ Late diagnosis is associated with chronic complications such as growth failure, organ damage, and increased mortality.^5^, ^6^, ^7^ Effective strategies to address the barriers to HIV diagnosis in children are urgently needed.^8^

**Research in context**

**Evidence before this study**

HIV testing rates in children exposed to HIV remains low in prevention of mother-to-child transmission programmes in sub-Saharan Africa, with many children being diagnosed late in childhood when they develop advanced disease. Due to the relatively low HIV prevalence among children and adolescents, targeted HIV testing services (HTS) approaches such as index-linked testing (HIV testing offered to children in the same household as people with HIV) might be most efficient and cost-effective. On April 1, 2020, we searched Medline for studies on index-linked HIV testing in children and adolescents, without restrictions on date, location, or language. Using the keywords “HIV testing”, “index”, “children”, and “adolescents”, we found five studies that had evaluated index-linked testing in children and adolescents (age 0–19 years). The studies were done in Cameroon, Kenya, Lesotho, and Malawi between 2016 and 2019. All of the studies reported higher HIV yield with index-linked testing than that obtained via routine HTS. Only one of the studies offered index-linked HIV testing in both rural and urban settings and only two studies offered index patients a choice of test location (facility-based or community-based). None of the five studies evaluated factors associated with uptake of testing.

**Added value of this study**

Our study is the first to compare the uptake of index-linked testing in children via health facility testing versus community-based testing by lay workers or caregivers in an HIV-prevalent setting. We evaluated preferred choice of HIV testing modality, and index patient and child factors associated with choice and uptake of testing. Our study shows that, although facility-based methods are more commonly chosen when initially proposed, probably because this approach is well known to the public, actual uptake of HTS is higher with community-based approaches. Community-based approaches might address some of the key barriers to facility-based testing, such as travel costs, distance to a clinic, particularly in rural areas, and suboptimal access due to restrictive opening hours of health facilities. Our study also shows that community HTS provided by lay workers and caregivers is feasible.

**Implications of all the available evidence**

To reach the UNAIDS 95-95-95 targets (95% of HIV-positive people aware of status, ART for 95% of those diagnosed, and viral suppression for 95% of those treated by 2030), HTS strategies will need to focus on hard-to-reach populations and address barriers that existing approaches have not been able to overcome. Community-based approaches might address such barriers but will require sensitisation and education of communities, and support for caregivers who test their children. Quality control, monitoring, and linkage to care for children who test positive will need to accompany these approaches.

Given the relatively low prevalence of HIV in children,^2^, ^3^ targeted HIV testing strategies, including the use of screening tools and index-linked testing, have been suggested to improve testing efficiency and potentially reduce costs.^9^ Index-linked testing, whereby an HIV test is offered to contacts of an index case (ie, an individual living with HIV), has been shown to increase HIV yield when used in children, but as with any other testing strategy, it relies on uptake by parents and carers.^9^, ^10^ The test location (eg, health facilities *vs* community-based settings) might influence uptake.^11^ Barriers to facility-based testing in children include inflexible facility working hours, user fees, distance to clinics, transport costs, and insufficient or overworked health-care providers.^12^ In view of these barriers, WHO recommended the use of lay workers in community settings in 2018 to reduce costs and increase uptake of HIV testing services (HTS).^13^ Index-linked testing of children, adolescents, and young adults (age 1–24 years) in community-based settings resulted in higher uptake than at health facilities in Malawi.^14^ In recent years, self-testing with oral mucosal transudate (OMT) has been implemented among adults in sub-Saharan Africa,^15^, ^16^ and an extension of this approach whereby caregivers test their children might also improve accessibility.^17^ Offering indexes a choice of different HTS delivery models acknowledges different preferences, and providing flexibility in the place and mode of HTS delivery might optimise uptake of index-linked HIV testing.

Zimbabwe has an early-onset, sustained, severe HIV epidemic, with an adult HIV prevalence of 13% in 2018.^3^ HIV prevalence in children (age 0–14 years) was 1·6% and in young people (age 15–24 years) was 4·4% in 2016.^3^, ^18^ Although PMTCT coverage in Zimbabwe is high, with 94% of HIV-positive pregnant women receiving ART, only 63% of infants born to HIV-positive mothers were tested within the first 2 months of birth in 2019.^3^

In the Bridging the Gap in HIV testing and care for children in Zimbabwe (B-GAP) study, we evaluated index-linked testing provided in health facilities and via two community-based approaches (namely testing by a trained lay worker and HIV testing by the caregiver at home using OMT), for children aged 2–18 years in rural and urban Zimbabwe. The aim of this study was to investigate the uptake and yield of index-linked testing and factors associated with its acceptance. We also investigated the choice of testing methods (ie, community-based vs facility-based testing) and factors associated with acceptance of community-based over facility-based index-linked HIV testing. We hypothesised that offering HTS to children living in households with an HIV-positive individual will result in a high yield of HIV diagnoses when compared with approaches that are not targeted, and that the option for community-based HTS might increase uptake compared with facility-based HTS alone.

## Methods

### Study design and participants

The B-GAP study was an interventional study without a control group done in Bulawayo, the second largest city in Zimbabwe, and Matabeleland South province, between Jan 29 and Dec 12, 2018. Matabeleland South borders Botswana and has the highest HIV prevalence (20·4%) in the country.^18^ Three rural primary health-care clinics in Mangwe district in Matabeleland South and six urban primary health-care clinics in Bulawayo were purposively selected on the basis of the number of patients registered for HIV care and distance to another facility.^18^

At each facility, all individuals attending for HIV care, regardless of ART status and time since diagnosis, were screened over 3 months to identify index cases. The national HIV programme provides a 3 month drug supply, therefore the majority of clinic attendees were anticipated to have undergone eligibility screening for study inclusion by 3 months. After screening and inclusion, a structured questionnaire collecting sociodemographic data, the number of children in the household, and their HIV status was administered by research assistants to index patients and data was entered on electronic tablets into Open Data Kit (version 1.11.1). Index patients were defined as consenting HIV-positive individuals who had at least one child aged 2–18 years living in their household (regardless of whether or not they were a biological parent) who was eligible for HTS (ie, had never had an HIV test or had a negative HIV test more than 6 months before screening). Children who were reported to have had an HIV-negative test more than 6 months ago were eligible for testing as index patients could have over-reported testing of children, older adolescents might have been sexually active, and some children might have been victims of sexual abuse.

Written informed consent for participating in the study was obtained from all eligible index patients in either English, Shona, or Ndebele when they were approached at the health facility. Index individuals aged younger than 18 years were included in the study if accompanied by an adult who could provide consent. At the point of testing of children either at the facility or in the community, verbal consent from the child's parent or legal guardian and child assent (for children aged <16 years) or direct verbal consent from the child (for children aged ≥16 years) for HIV testing was sought.

Ethical approval for this study was obtained from the Medical Research Council of Zimbabwe, the institutional review board of the Biomedical Research and Training Institute of Zimbabwe, and the London School of Hygiene & Tropical Medicine ethics committee. The B-GAP study protocol has been published previously.^19^

### Procedures

Index patients were offered three choices of HIV testing for eligible children living in their household: testing in the health facility; testing by a lay worker at home; or testing by the index caregiver at home. Testing in the facility was done by routine clinic staff or research assistants. Research assistants were the lay workers and in addition to training for the study had undergone a 2-week training course on rapid HIV testing and counselling provided by a private training institution affiliated with the Zimbabwe Ministry of Health and Child Care. The lay workers were stationed at each clinic. Index patients could only elect for caregiver testing if they were a biological parent or the legal guardian to ensure safeguarding of children who would be tested in the absence of a health-care provider. Caregiver testing was done with OMT (OraQuick ADVANCE Rapid HIV-1/2; OraSure Technologies, Bethlehem, PA, USA). Caregivers were counselled and shown how to do the test by research assistants at each facility. If assessed by the research assistant to be competent to test, they were given an OMT HIV test for each eligible child and asked to do the test within 5 days. A helpline number for counselling and support was provided. Results of caregiver-provided testing were by self-report. Index patients who chose caregiver-provided testing were told to return to the facility for confirmatory HIV testing in the event of a reactive OMT test as per WHO guidelines.^20^

Index patients who chose community testing by a lay worker were visited at home on a scheduled date. Up to two further home visits were undertaken if the child was not present on the scheduled date. For facility and caregiver-provided testing, all index patients were contacted by lay workers by phone on days 7, 14, and 21 post-screening if their children had not yet been tested. If unreachable by phone, a maximum of two home visits were undertaken and HIV testing done at home by a lay worker if the index patient and child consented. Therefore, index patients who initially chose facility-based testing could later consent to testing in the home. Similarly, index patients who initially chose community-based testing could, at a later date, switch to testing at the facility. The test method chosen by the index patient at screening and the final test location for children was recorded. Testing at the facility or community-based testing by a lay worker was done according to the national HIV testing algorithm via a blood-based rapid diagnostic test, with results available on the same day.^21^ We defined community-based testing as either testing done by a lay worker at home or caregiver-provided testing.

Children were given age-appropriate explanations of the test results, determined by the age and maturity of the child and guided by the Zimbabwe Ministry of Health and Child Care guidelines for HIV testing and counselling for children.^22^

For children who were not tested, the reason was recorded. Children who tested HIV-positive were referred to their nearest facility or the preferred facility of the child or household for onward care.

### Outcomes

The primary outcome of the study was the uptake of testing, defined as the proportion of eligible children having an HIV test during the study. Secondary outcomes were the uptake of the different HIV testing methods, HIV yield, and HIV prevalence. HIV yield was defined as the proportion of HIV-positive children among all eligible children. HIV prevalence was defined as the proportion of HIV-positive children among those tested. Any children with an unknown test outcome were classed as not tested and therefore not included in the denominator for HIV prevalence.

### Statistical analysis

Sample size estimations were based on precision of the estimated proportion taking up HIV testing. An average of 32 clients were anticipated to attend a clinic per day. A sample size of 6739 children would provide a 1% precision interval for an estimate of 80% of children taking up testing. An average of 32 clients were anticipated to attend a clinic per day based on routine data on attendance of each clinic in 2017. Therefore, during the 3 month study period at each clinic, in nine clinics assuming 5 working days per week, this visit rate would provide sufficient numbers of index patients assuming 30–40% of index patients had eligible children and accepted participation. Within-index clustering was not taken into account when calculating sample size due to the small numbers of children expected in each household.

All analyses were done with Stata software (version 15.0). Continuous variables were summarised as means and SDs or medians and IQRs, and categorical variables as counts and percentages. We used univariable logistic regression to investigate the association between index patient characteristics and at least one child in their household having an HIV test, and between child characteristics and a child having an HIV test. Index characteristics of interest were age, sex, health-care facility setting (rural or urban), highest level of education, time since HIV diagnosis, mode of transport to the facility, and cost of travel to the facility. Child characteristics of interest were age, sex, HIV status, relationship to index patient, orphanhood status, mother's HIV status, history of receiving ART for PMTCT, and any previous offer of HIV testing. For each model, significant index patient or child variables (at p<0·10) in univariable analysis were retained in multivariable logistic regression models (significance deemed at p<0·10). Using logistic regression we also evaluated the odds of having an HIV test by selected testing model adjusted for clustering by index. For models including child characteristics, robust standard errors or generalised estimating equations were used to allow for household-level clustering. Additionally, index patient and child characteristics associated with selection of community-based testing models versus facility-based testing at screening were evaluated in univariable and multivariable models. As sensitivity analyses, we investigated factors associated with selection of testing by a lay worker versus facility-based testing, and with selection of testing provided by a caregiver versus facility-based testing. Clustering by index patient was adjusted for in all models including child variables. An independent project steering committee met annually.

### Role of the funding source

The funders of the study had no role in study design, data collection, data analysis, data interpretation, or writing of the report. The corresponding author had full access to all the data in the study and had final responsibility for the decision to submit for publication.

## Results

**Figure 1:**
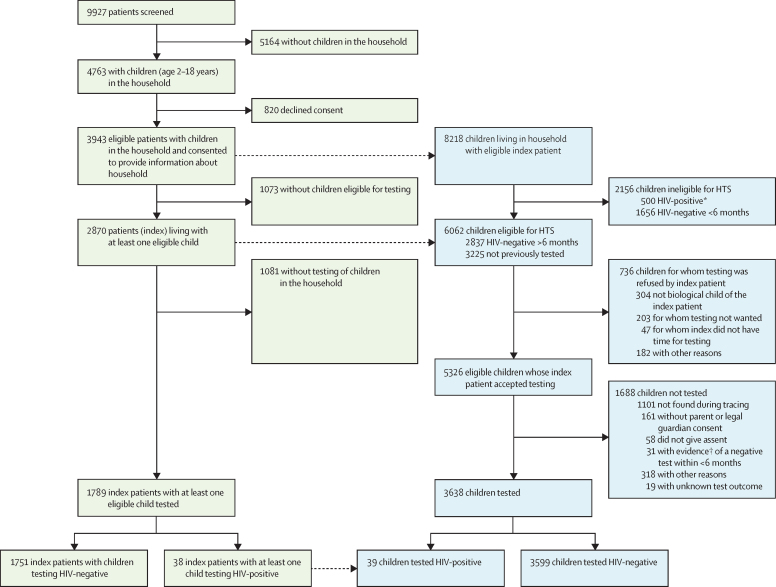
Screening and HIV testing flow for HIV-positive clinic attendees (green) and children living in their households (blue) HTS=HIV testing services. *490 children registered in care and 10 not registered in care. †Test result slip or note in the child's medical records.

Between Jan 29 and Dec 12, 2018, 9927 individuals were screened in the nine primary health-care clinics (427–2005 per clinic), of whom 5164 reported no children aged 2–18 years in the household and 820 declined consent to provide information about children in their household. Of the remaining 3943 individuals, 2870 had at least one child eligible for HTS in their household and were therefore classed as index patients (figure 1). The median age of index patients was 39 years (IQR 32–46) and 2259 (78·8% of 2866 with available data) were women. 1622 (56·5%) of the 2870 index patients had been diagnosed with HIV in the past 5 years. The main means of transport to the clinic was by foot (for 1874 [65·4%] of 2866 with available data; table 1). The median number of children living in the households of index patients was 1 (IQR 1–3).

**Table 1:** Index patient characteristics associated with HIV testing in at least one child

	**Index patient population**		**Univariable analysis**		Multivariable analysis*	
	Patients with at least one eligible child (n=2870), n (%)	Patients with at least one child tested (n=1789), n (% of those with ≥1 eligible child)	OR (95% CI)	p value	aOR (95% CI)	p value
**Age, years**						
0–18	54 (1·9%)	36 (66·7%)	1·00 (ref)	..	..	..
19–34	892 (31·1%)	566 (63·5%)	0·87 (0·49–1·55)	..	..	..
35–59	1767 (61·6%)	1087 (61·5%)	0·80 (0·45–1·42)	..	..	..
≥60	157 (5·5%)	101 (64·3%)	0·90 (0·47–1·73)	0·63	..	..
**Sex**†						
Male	607 (21·2%)	329 (54·2%)	1·00 (ref)	..	1·00 (ref)	..
Female	2259 (78·8%)	1456 (64·5%)	1·53 (1·28–1·84)	<0·0001	1·56 (1·38–1·77)	<0·0001
**Site**						
Rural	786 (27·4%)	504 (64·1%)	1·00 (ref)	..	..	..
Urban	2084 (72·6%)	1286 (61·7%)	0·90 (0·76–1·07)	0·24	..	..
**Highest level of education completed**†						
None	45 (1·6%)	27 (60·0%)	1·00 (ref)	..	..	..
Primary	909 (31·7%)	571 (62·8%)	1·13 (0·61–2·08)	..	..	..
Secondary	1817 (63·4%)	1136 (62·5%)	1·11 (0·61–2·03)	..	..	..
Tertiary	95 (3·3%)	52 (54·7%)	0·77 (0·38–1·59)	0·36	..	..
**Mode of transport to facility**†‡						
By foot	1874 (65·4%)	1259 (67·2%)	1·00 (ref)	..	..	..
Public transport	844 (29·4%)	442 (52·4%)	0·54 (0·46–0·64)	<0·0001	..	..
By car	42 (1·5%)	20 (47·6%)	0·45 (0·24–0·82)	0·0097	..	..
Other	106 (3·7%)	65 (61·3%)	0·78 (0·52–1·16)	0·22	..	..
**Cost of travel to facility**‡						
No cost (0 US$)	1998 (69·6%)	1332 (66·7%)	1·00 (ref)	..	1·00 (ref)	..
Some cost (>0 US$)	872 (30·4%)	458 (52·5%)	0·86 (0·83–0·90)	<0·0001	0·86 (0·83–0·88)	<0·0001
**Time since HIV diagnosis, years**§						
<1	290 (10·2%)	174 (60·0%)	1·00 (ref)	..	..	..
1–5	1332 (46·6%)	820 (61·6%)	1·06 (0·82–1·38)	..	..	..
6–10	990 (34·7%)	618 (62·4%)	1·10 (0·85–1·45)	..	..	..
>10	245 (8·6%)	168 (68·6%)	1·45 (1·02–2·08)	0·16	..	..

Tested children were classed as all those who received a test and had a known test outcome**.** OR=odds ratio. aOR=adjusted OR**.**

* Significant index patient variables (at p <0·10) in univariable analysis were retained in multivariable logistic regression.

† Missing data for four index patients.

‡ Only cost of travel to facility included in multivariate analysis due to collinearity with mode of transport to facility.

§ Missing data for 13 index patients.

8218 children (4147 [50·5%] girls [sex unknown in nine], median age 9 years [IQR 5–13]) living in the households of index patients (n=3943) were screened for eligibility (figure 1). Of these children, 6062 (73·8%) were eligible for index-linked testing (3115 [51·4%] girls [sex unknown in seven], median age 8 years [IQR 5–13]), with 2837 (34·5%) having tested HIV-negative more than 6 months ago, and 3225 (39·2%) having not previously tested. The range in eligibility prevalence by clinic was 54·4–98·6%. 500 (6·1%) children had a known HIV-positive status.

Of the 2870 index patients with at least one eligible child, 1789 (62·3%) had at least one child in their household tested, including 1012 (35·3%) who had two or more children tested. Overall, 1476 (51·4%) index patients had all eligible children in their households tested. In terms of corresponding numbers in children, HIV testing was accepted for 5326 (87·9%) of 6062 eligible children (range by clinic 61·3–98·4%). The main reason for index patients refusing testing in 736 children was that the index patient was not the biological parent of the eligible child (304 [41·3%] children). In the 5326 children with acceptance for testing, 3638 (68·3%) were subsequently tested (range by clinic 53·5–92·0%), representing an overall uptake of HTS among 6062 eligible children of 60·0% (figure 1). Among 1688 children not tested despite acceptance by the index patient, the main reasons for non-uptake were inability to contact children (1101 [65·2%]) and no consent from guardians at the point of testing (161 [9·5%]).

Of the 3638 children with an HIV test outcome, 39 were positive, giving an HIV prevalence of 1·1%, and an HIV yield among 6062 eligible children of 0·6%. HIV prevalence was 1·0% (24 of 2322 children) in urban settings and 1·1% (15 of 1316) in rural settings. The median age of children diagnosed with HIV was 11 years (IQR 8–15; range 3–18) and 28 (71·8%) were girls. 17 (43·6%) of the 39 children were single or double orphaned, 25 (64·1%) were biological children of the index patient, 28 (71·8%) had not been previously tested, and nine (23·1%) were linked to index patients who had been diagnosed within the past year. HIV was diagnosed in 26 (prevalence 1·4%) of 1916 children tested in a health-care facility and 13 (0·8%) of 1722 children tested in the community. In the community, HIV was diagnosed in 12 (0·8%) of 1522 children tested by a lay worker, and one (0·5%) of 200 tested by their caregiver using OMT and confirmed HIV-positive at their health-care facility. Of the 39 children who tested HIV-positive and received a referral, 36 (92·3%) were registered with a facility.

The proportion of index patients who had at least one child tested was similar between urban (1286 [61·7%] of 2084 index patients) and rural (504 [64·1%] of 786) sites (table 1). In our univariable analysis of index patient characteristics, female sex, cost to travel to the facility, and mode of transport were associated with HIV testing for at least one child in the household. In multivariable analysis, female sex of the index patient was associated with at least one child in the household having an HIV test (adjusted odds ratio [aOR] 1·56 [95% CI 1·38–1·77], p<0·0001). Additionally, children were less likely to be tested if the index patient reported any financial cost for them to travel to the facility (aOR 0·86 [0·83–0·88], p<0·0001; table 1).

In our univariable analysis of child characteristics, having an HIV test was associated with sex, age, HIV status, relationship to the index patient, and mother's HIV status. In multivariable analysis, female sex of the child was associated with having an HIV test (aOR 1·10 [95% CI 1·03–1·19], p=0·0080). Increased age of the child (6–9 years: aOR 0·99 (0·89–1·09); 10–15 years: aOR 0·91 [0·83–1·00]; and 16–18 years: aOR 0·75 [0·66–0·85]; p=0·0001 *vs* 2–5 years) and unknown HIV status of the mother (aOR 0·81 [0·68–0·98], p=0·027 *vs* HIV-positive status) were associated with reduced odds of having an HIV test (table 2).

**Table 2:** Child characteristics associated with having an HIV test

	**Child population**		Univariable analysis*		Multivariable analysis (n=5043)*†	
	Eligible children (n=6062), n (%)	Children tested (n=3638), n (% of those eligible)	aOR (95% CI)	p value	aOR (95% CI)	p value
**Sex**‡						
Male	2940 (48·6%)	1720 (58·5%)	1·00 (ref)	..	1·00 (ref)	..
Female	3115 (51·4%)	1911 (61·3%)	1·07 (1·01–1·15)	0·032	1·10 (1·03–1·19)	0·0080
**Age, years**‡						
2–5	1801 (29·7%)	1159 (64·4%)	1·00 (ref)	..	1·00 (ref)	..
6–9	1586 (26·2%)	956 (60·3%)	0·97 (0·89–1·06)	..	0·99 (0·89–1·09)	
10–15	1981 (32·7%)	1174 (59·3%)	0·89 (0·82–0·97)	..	0·91 (0·83–1·00)	
16–18	687 (11·3%)	342 (49·8%)	0·73 (0·65–0·82)	<0·0001	0·75 (0·66–0·85)	0·0001
**HIV status**§						
Never tested	3224 (53·2%)	1930 (59·9%)	1·00 (ref)	..	1·00 (ref)	..
Known negative >6 months	2837 (46·8%)	1708 (60·2%)	1·10 (1·02–1·20)	0·021	1·06 (0·96–1·17)	0·28
**Relationship to index patient**‡						
Non-biological child	2482 (41·0%)	1466 (59·1%)	1·00 (ref)	..	1·00 (ref)	..
Biological child	3573 (59·0%)	2165 (60·6%)	1·22 (1·11–1·33)	<0·0001	1·12 (0·96–1·32)	0·16
**Orphanhood status**‡						
Not orphaned	4728 (78·1%)	2796 (59·1%)	1·00 (ref)	..	..	..
Paternal orphan	960 (15·9%)	613 (63·9%)	1·00 (0·90–1·12)	0·98	..	..
Maternal orphan	190 (3·1%)	101 (53·2%)	0·98 (0·79–1·23)	0·88	..	..
Double orphan	177 (2·9%)	121 (68·4%)	1·15 (0·91–1·46)	0·23	..	..
**Mother's HIV status**¶						
HIV-positive	3223 (63·9%)	2048 (63·5%)	1·00 (ref)	..	1·00 (ref)	..
HIV-negative	901 (17·9%)	585 (64·9%)	0·83 (0·74–0·94)	0·0037	0·90 (0·76–1·07)	0·24
Unknown to index patient	920 (18·2%)	475 (51·6%)	0·73 (0·64–0·83)	<0·0001	0·81 (0·68–0·98)	0·027
**Prevention of mother-to-child transmission treatment history**‖						
No	1839 (55·4%)	1147 (62·4%)	1·00 (ref)	..	..	..
Yes	1284 (38·7%)	758 (59·0%)	1·07 (0·97–1·18)	0·20	..	..
Unknown to index patient	196 (5·9%)	115 (58·7%)	1·05 (0·83–1·33)	0·67	..	..
**Previous offer for HIV testing**‖						
No	1191 (35·9%)	715 (60·0%)	1·00 (ref)	..	..	..
Yes	1999 (60·2%)	1216 (60·8%)	1·05 (0·95–1·17)	0·36	..	..
Unknown to index patient	136 (4·1%)	96 (70·6%)	1·10 (0·82–1·49)	0·52	..	..

Tested children were classed as all those who received a test and had a known test outcome. aOR=adjusted odds ratio (with adjustment for clustering by index patient).

* Logistic regression with generalised estimating equations.

† Significant child variables (at p <0·10) in univariable analysis were retained in multivariable logistic regression; n reflects children tested minus those with data missing on model variables.

‡ Missing data for seven children.

§ Missing data for one child.

¶ Missing data for 1018 children as question was introduced into the study after March 1, 2018.

‖ Question only asked if the child was the biological child of the index patient (n=3319).

**Figure 2:**
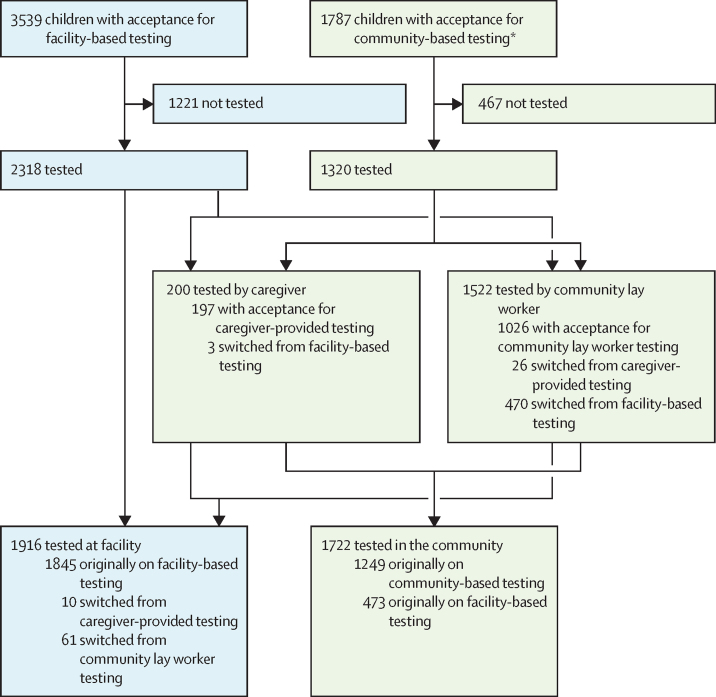
Selection of facility-based (blue) and community-based (green) HIV testing for eligible children whose index patient accepted testing *1487 children with acceptance for testing by a lay worker and 300 for testing by a caregiver at screening.

Of the 5326 eligible children whose index patient accepted testing at screening, facility-based testing was chosen for 3539 (66·4%) children and community-based testing for 1787 (33·6%; figure 2). Per clinic, the proportion of children whose index patients opted for community-based testing ranged from 8·9% to 81·1%. In univariable analysis adjusting for clustering by index patient, the odds of the child being tested were higher if the index patient selected community-based testing versus facility-based testing (1320 [73·9%] children tested of 1787 *vs* 2318 [65·5%] of 3539; aOR 1·49 [95% CI 1·22–1·81], p=0·0001).

Regardless of initial choice, similar proportions of eligible children were tested by facility-based and community-based approaches (of 3638 children with a known test outcome overall, 1916 [52·7%] were tested at a facility *vs* 1722 [47·3%] in the community). 2318 children with initial acceptance for facility-based testing went on to be tested, 473 (20·4%) of whom were tested in the community. By contrast, 1320 children with acceptance for community-based testing went on to be tested, of whom 71 (5·4%) were tested in a facility (figure 2).

In univariate analysis adjusted for clustering by index, factors associated with uptake of community-based HIV testing were site type (urban or rural), index patient age, cost of travel to a facility, time since diagnosis in the index patient, child age, child HIV status, PMTCT treatment history, and a previous offer of HIV testing. On multivariable analysis, urban residence (aOR 2·29 [95% CI 1·80–2·91], p<0·0001), any travel cost to the facility (aOR 1·21 [1·15–1·28], p<0·0001), and time since HIV diagnosis in the index patient (1–5 years since diagnosis: aOR 1·58 [1·07–2·31]; and ≥6 years since diagnosis, aOR 1·59 [1·07–2·35]; p=0·070 *vs* diagnosis <1 year ago) were associated with selection of community-based testing compared with facility-based testing at screening (table 3). Increased child age (6–9 years: aOR 1·20 [1·02–1·41]; and 10–15 years: aOR 1·35 [1·14–1·59]; p=0·0020 *vs* 2–5 years) and unknown HIV status in the child (aOR 1·33 [1·12–1·59], p=0·0013) were also associated with selection of community-based testing at screening. Results were similar in our sensitivity analysis, in which the outcome was defined as testing delivered by a community lay worker (n=1487 children) compared with facility-based testing (appendix pp 1, 2). Selection of caregiver-provided testing (n=300 children) versus facility-based testing at screening was associated with urban residence, the cost of transport, unknown HIV status in the child, and, additionally, female sex of the index patient (appendix pp 3, 4). No adverse events were reported in our study.

**Table 3:** Factors associated with uptake of community-based testing options at screening

		**Community-based testing chosen (n=1787 children)**	**Univariable analysis**		Multivariable analysis*(n=5262)	
			aOR (95% CI)	p value	aOR (95% CI)	p value
**Index patient variables**						
Age, years						
	0–18	29 (1·6%)	1·00 (ref)	..	1·00 (ref)	..
	19–34	480 (26·9%)	1·48 (0·63–3·47)	..	1·07 (0·46–2·45)	..
	35–59	1146 (64·1%)	1·99 (0·86–4·60)	..	1·27 (0·56–2·89)	..
	≥60	132 (7·4%)	2·32 (0·92–5·88)	0·02	1·64 (0·65–4·12)	0.22
Sex						
	Male	363 (20·3%)	1·00 (ref)	..	..	..
	Female	1424 (79·7%)	1·00 (0·79–1·27)	0·98	..	..
Site						
	Rural	448 (25·1%)	1·00 (ref)	..	1·00 (ref)	..
	Urban	1339 (74·9%)	2·51 (1·99–3·17)	<0·0001	2·29 (1·80–2·91)	<0·0001
Highest level of education						
	None	33 (1·9%)	1·00 (ref)	..	..	..
	Primary	538 (30·1%)	0·74 (0·33–1·63)	0·45	..	..
	Secondary	1143 (64·0%)	1·19 (0·54–2·58)	0·67	..	..
	Tertiary	73 (4·1%)	1·90 (0·76–4·70)	0·17	..	..
Mode of transport to facility†						
	By foot	1003 (56·1%)	1·00 (ref)	..	..	..
	Public transport	691 (38·7%)	2·42 (1·95–3·00)	<0·0001	..	..
	By car	12 (0·7%)	0·64 (0·25–1·65)	0·35	..	..
	Other	81 (4·5%)	1·01 (0·60–1·68)	0·98	..	..
Cost of travel to facility, US$†						
	No cost (0 US$)	1090 (61·0%)	1·00 (ref)	..	1·00 (ref)	..
	Some cost (>0 US$)	697 (39·0%)	1·24 (1·18–1·31)	<0·0001	1·21 (1·15-1·28)	<0·0001
Time since HIV diagnosis, years‡						
	<1	132 (7·5%)	1·00 (ref)	..	1·00 (ref)	..
	1–5	815 (46·5%)	1·53 (1·07–2·20)	..	1·58 (1·07–2·31)	..
	≥6	804 (45·9%)	1·48 (1·03–2·13)	0·066	1·59 (1·07–2·35)	0·070
**Child variables**						
Sex§						
	Male	884 (49·5%)	1·00 (ref)	..	..	..
	Female	902 (50·5%)	0·95 (0·84–1·07)	0·42	..	..
Age, years§						
	2–5	472 (26·4%)	1·00 (ref)	..	1·00 (ref)	..
	6–9	474 (26·5%)	1·26 (1·09–1·47)	..	1·20 (1·02–1·41)	..
	10–15	645 (36·1%)	1·41 (1·21–1·63)	..	1·35 (1·14–1·59)	..
	16–18	195 (10·9%)	1·23 (0·99–1·53)	0·0001	1·08 (0·86–1·38)	0·0020
HIV status§¶						
	Known HIV negative >6 months	1028 (57·6%)	1·00 (ref)	..	1·00	..
	Unknown	758 (42·4%)	1·32 (1·17–1·48)	<0·0001	1·33 (1·12–1·59)	0·0013
Relationship to index§						
	Non-biological child	672 (37·6%)	1·00 (ref)	..	..	..
	Biological child	1114 (62·4%)	1·09 (0·94–1·26)	0·24	..	..
Orphanhood status§						
	Not orphaned	1387 (77·7%)	1·00 (ref)	..	..	..
	Paternal orphan	288 (16·1%)	0·96 (0·78–1·19)	0·71	..	..
	Maternal orphan	45 (2·5%)	0·77 (0·48–1·23)	0·28	..	..
	Double orphan	66 (3·7%)	1·28 (0·86–1·93)	0·23	..	..
Mother's HIV status‖						
	HIV-positive	985 (65·9%)	1·00 (ref)	..	..	..
	HIV-negative	247 (16·5%)	0·81 (0·63–1·05)	0·11	..	..
	Unknown to index	262 (17·5%)	1·14 (0·88–1·48)	0·31	..	..
Prevention of mother-to-child transmission treatment history**††						
	Yes	344 (32·2%)	1·00 (ref)	..	..	..
	No	646 (60·4%)	1·26 (1·05–1·52)	0·015	..	..
	Unknown to index	80 (7·5%)	2·32 (1·50–3·59)	0·0002	..	..
Previous offer for HIV testing¶**						
	Yes	577 (53·9%)	1·00 (ref)	..	..	..
	No	443 (41·4%)	1·48 (1·21–1·81)	0·0002	..	..
	Unknown to index	50 (4·7%)	1·40 (0·84–2·33)	0·20	..	..

**aOR**=adjusted odds ratio (with adjustment for clustering by index patient).

* Significant index patient variables (at p<0·10) in univariable analysis were retained in multivariable logistic regression; n reflects children tested minus those with data missing on model variables.

† Only cost of travel to facility included in multivariate analysis due to collinearity with mode of transport to facility.

‡ Missing data for 36 index patients.

§ Missing data for one child.

¶ Only HIV status included in multivariable analysis due to collinearity with previous offer for HIV testing.

‖ Missing data for 293 children as question was introduced into the study after March 1, 2018.

** Question only asked if the child was the biological child of the index patient (n=1070).

†† Excluded in multivariable model due to high number of missing data.

## Discussion

We found an overall uptake of index-linked HIV testing of 60% after offering index patients a choice of facility-based or community-based HTS. A greater proportion of index patients chose facility-based testing over community-based testing; however, children were more likely to be tested if the index chose community-based testing. Index patients who had to pay to get to the clinic were less likely to have children in their households tested, and older children were less likely to be tested. Some index patients refused testing for children because they were not the biological parents of children living in their households. This finding highlights the challenges of identifying children with HIV, who are disproportionately likely to be orphaned.^23^ Although being a biological child was associated with having a test in univariable analysis, this effect was not significant in multivariate analysis. We also found that female index patients were more likely to have at least one child tested and female children were more likely to be tested than their male counterparts. Poor health-seeking behaviour is commonly reported among men and our study highlights the need for further work to engage men, to inform the development of acceptable HTS.^24^

The yield of HIV was lower than anticipated when compared with blanket approaches such as outpatient and inpatient provider-initiated testing and counselling in Zimbabwe and within sub-Saharan Africa, in which HIV yield among children and adolescents ranged from 7·4% to 12·2%.^11^ This finding might be due to the scale-up of the PMTCT programme in Zimbabwe, where coverage was 94% in 2018.^3^, ^25^ Although index-linked testing implemented in Kenya and Malawi detected high prevalence of HIV (7·4% among children aged <12 years in Kenya and 4·0% among individuals aged 1–24 years in Malawi), their PMTCT coverage was similar at 91% and 95%, indicating the possibility of missed diagnoses before the scale-up of PMTCT.^3^, ^14^, ^26^ Alternatively, low yield could also imply that even index-linked HTS is an insufficient strategy to address the gap in HIV testing for children in Zimbabwe. Some index patients refused testing or did not complete testing for eligible children in their households. Further studies evaluating reasons or risk factors for non-uptake of testing are therefore warranted. However, we do note that while 500 children living in households with an index patient were known to be HIV-positive before the study, 39 (7·2%) of 539 children (500 who were known HI-positive and 39 newly diagnosed in this study) had been missed by existing services.

The median age of children diagnosed with HIV in our study was 11 years. Older children are at particularly high risk of living with undiagnosed HIV as they have missed HIV testing within PMTCT programmes^27^ and were less likely to be tested in our study. A misplaced assumption might be that an older child who is not ill is unlikely to have perinatally acquired HIV infection, particularly when the mother's status is not known. In addition, adolescents are a challenging group to engage within health services. In our study, older children and children whose mother's HIV status was not known had lower odds of being tested. Our findings highlight the need for continued efforts to expand HTS particularly among older adolescents. Notably, this age group is also at risk of horizontal transmission and index-linked testing might need to be combined with other approaches.

Facility-based index-linked testing has been recommended in WHO guidelines and in Zimbabwean national guidelines.^19^, ^28^ Our study found that similar proportions of children were tested by facility-based or community-based index-linked HIV testing, despite substantially more index patients choosing facility-based testing at screening. Community-based testing potentially addresses some of the barriers to HTS, such as costs of travel to health facilities and the time taken by index patients to bring children to facilities. Children were less likely to be tested if the index patient had to pay for travel (eg, via public transport or a car) rather than walk to a health facility. In addition, paying for travel to a health facility was associated with selection of community-based rather than facility-based HIV testing by the index patient. Community-based index-linked testing might identify children who are particularly hard to reach with currently available approaches. Indeed, index patients who had been diagnosed with HIV for a long period of time, increased age of the child, and no history of previous testing of the child, all of which are indicators of a lack of engagement with HTS for children, were independently associated with selection of community-based HIV testing.

Although WHO has recommended community-based HIV testing by lay workers,^13^ in many countries including Zimbabwe, HTS in the community is done by nurses. In the B-GAP study, lay workers underwent 2 weeks of training on study processes and rapid HIV testing and caregivers were also able to test their children using an oral HIV test after brief demonstrations within the health facility. Our study shows the feasibility of lay individuals with minimal training implementing community-based HTS. HIV self-testing for adults has had high uptake among groups such as men, older adolescents (age 16–18 years), sex workers, and men who have sex with men.^13^, ^15^, ^16^ This high uptake is often attributed to ease, privacy, and confidentiality, and the non-invasiveness of the test compared with blood-based testing.^13^, ^15^, ^29^ We identified a low uptake of caregiver-provided testing, probably due to poor awareness of self-testing among our study population, for whom HIV self-testing is not yet routinely or widely available. In our study, female index patients were more likely to take up caregiver-provided testing, which might reflect the potential usefulness of this strategy within PMTCT programmes.

Community-based testing by lay workers or caregivers could reduce workload for facility-based health workers and has potential to be cost-effective.^30^ Additionally, community-based testing could be used as a back-up mechanism for children who do not present for facility-based index-linked testing. Many children in our study were subsequently tested in the community, as lay workers visited children at home who did not attend facilities for testing as per the protocol procedures.^22^ Notably, 20% of children whose index patients initially opted for facility-based testing were subsequently tested by a community-based approach, but only 5% whose index patients opted for community-based testing were ultimately tested in a health facility.

The strengths of this study include a large sample size and comprehensive ascertainment of outcomes. The inclusion criteria were not restricted to biological children of index patients to ensure that children who might have been orphaned were not excluded. The study was done in public sector clinics and in both urban and rural settings, making the findings generalisable to similar settings within Zimbabwe and the sub-Saharan Africa region. A limitation of our approach, whereby the starting point of offering testing is at the facility level, is that it excludes index patients who are disengaged from care and whose children might be HIV-positive but without a confirmed diagnosis. Additionally, contacting participants to ascertain outcomes might have indirectly increased uptake. Furthermore, oral testing results were obtained by self-report. We did a substudy to investigate accuracy of caregiver testing and findings will be reported separately. A further limitation was that the study was not aimed or powered to investigate index patient factors associated with an HIV-positive child diagnosis. If completed, a study focused on index factors associated with HIV-positive child status would allow health service providers to further target index-linked testing in children and adolescents, and we therefore recommend future studies on this aspect.

Evaluation of the affordability of index-linked approaches is crucial to inform scalability. Previous studies have shown that community-based HTS strategies among adults, including HIV self-testing, are cost-effective compared with facility based HTS in South Africa and Malawi.^31^, ^32^ However, cost-effectiveness of any approach will depend on HIV yield and uptake of HTS. A cost evaluation of the HTS approaches in the B-GAP study is underway and will be reported separately.

Substantial progress has been made in reducing the incidence of HIV infection, due to the scale-up of PMTCT programmes and improved coverage of ART in children. However, implementing strategies to identify hard-to-reach groups of children is imperative if the UNAIDS 95-95-95 targets (95% of HIV-positive people aware of status, ART for 95% of those diagnosed, and viral suppression for 95% of those treated by 2030) are to be met. Increasing the reach of testing will require strategies that target residual barriers to accessing HIV testing, which approaches to date have not been able to overcome. Although a targeted approach such as index-linked testing is an efficient approach in children in whom HIV prevalence is low, coverage to date has not been optimal. Our study provides evidence for the effectiveness of community-based approaches via lay health workers and caregivers for the testing of children at risk of HIV. Community-based testing might reduce burden on health facilities and address barriers to HTS access. Combined approaches and providing index patients with choice and flexibility might further improve uptake.

## Data sharing

The B-GAP dataset is publicly available from the London School of Hygiene & Tropical Medicine repository, Data Compass.

## References

[1] Violari A, Cotton MF, Gibb DM (2008). Early antiretroviral therapy and mortality among HIV-infected infants. N Engl J Med.

[2] UNAIDS (2020). Global HIV and AIDS statistics—2020 factsheet. https://www.unaids.org/sites/default/files/media_asset/UNAIDS_FactSheet_en.pdf.

[3] UNAIDS (2019). UNAIDS data 2019. https://www.unaids.org/sites/default/files/media_asset/2019-UNAIDS-data_en.pdf.

[4] Simms V, Dauya E, Dakshina S (2017). Community burden of undiagnosed HIV infection among adolescents in Zimbabwe following primary healthcare-based provider-initiated HIV testing and counselling: a cross-sectional survey. PLoS Med.

[5] Gregson CL, Hartley A, Majonga E (2019). Older age at initiation of antiretroviral therapy predicts low bone mineral density in children with perinatally-infected HIV in Zimbabwe. Bone.

[6] Majonga ED, Rehman AM, Simms V (2018). High prevalence of echocardiographic abnormalities in older HIV-infected children taking antiretroviral therapy. AIDS.

[7] Lowenthal ED, Bakeera-Kitaka S, Marukutira T, Chapman J, Goldrath K, Ferrand RA (2014). Perinatally acquired HIV infection in adolescents from sub-Saharan Africa: a review of emerging challenges. Lancet Infect Dis.

[8] Bandason T, McHugh G, Dauya E (2016). Validation of a screening tool to identify older children living with HIV in primary care facilities in high HIV prevalence settings. AIDS.

[9] Yumo HA, Kuaban C, Ajeh RA (2018). Active case finding: comparison of the acceptability, feasibility and effectiveness of targeted versus blanket provider-initiated-testing and counseling of HIV among children and adolescents in Cameroon. BMC Pediatr.

[10] Jubilee M, Park FJ, Chipango K, Pule K, Machinda A, Taruberekera N (2019). HIV index testing to improve HIV positivity rate and linkage to care and treatment of sexual partners, adolescents and children of PLHIV in Lesotho. PLoS One.

[11] Govindasamy D, Ferrand RA, Wilmore SMS (2015). Uptake and yield of HIV testing and counselling among children and adolescents in sub-Saharan Africa: a systematic review. J Int AIDS Soc.

[12] Dziva Chikwari C, Dringus S, Ferrand RA (2018). Barriers to, and emerging strategies for, HIV testing among adolescents in sub-Saharan Africa. Curr Opin HIV AIDS.

[13] WHO (October, 2018). HIV self-testing strategic framework. A guide for planning, introducing and scaling up. https://www.afro.who.int/sites/default/files/2019-12/9789241514859-eng.pdf.

[14] Ahmed S, Sabelli RA, Simon K (2017). Index case finding facilitates identification and linkage to care of children and young persons living with HIV/AIDS in Malawi. Trop Med Int Health.

[15] Choko AT, MacPherson P, Webb EL (2015). Uptake, accuracy, safety, and linkage into care over two years of promoting annual self-testing for HIV in Blantyre, Malawi: a community-based prospective study. PLoS Med.

[16] Indravudh PP, Choko AT, Corbett EL (2018). Scaling up HIV self-testing in sub-Saharan Africa: a review of technology, policy and evidence. Curr Opin Infect Dis.

[17] Dziva Chikwari C, Njuguna IN, Neary J (2019). Brief report: diagnostic accuracy of oral mucosal transudate tests compared with blood-based rapid tests for HIV among children aged 18 months to 18 years in Kenya and Zimbabwe. J Acquir Immune Defic Syndr.

[18] Ministry of Health and Child Care Zimbabwe (August, 2019). Zimbabwe population-based HIV impact assessment (ZIMPHIA) 2015–2016 final report. https://phia.icap.columbia.edu/wp-content/uploads/2019/08/ZIMPHIA-Final-Report_integrated_Web-1.pdf.

[19] Dziva Chikwari C, Simms V, Dringus S (2019). Evaluating the effectiveness and cost-effectiveness of health facility-based and community-based index-linked HIV testing strategies for children: protocol for the B-GAP study in Zimbabwe. BMJ Open.

[20] WHO (July, 2015). Consolidated guidelines on HIV testing services. https://www.who.int/hiv/pub/guidelines/hiv-testing-services/en/.

[21] Ministry of Health and Child Care Zimbabwe (May, 2014). Zimbabwe National Guidelines in HIV Testing and Counselling. https://hivstar.lshtm.ac.uk/files/2016/06/ZIMBABWE-National-Guidlines-on-HTC-2014.compressed.pdf.

[22] Ministry of Health and Child Care Zimbabwe (2008). HIV testing and counselling for children: a training course for counsellors. https://www.thecompassforsbc.org/project-examples/hiv-testing-and-counselling-children-training-course-counsellors.

[23] Birdthistle IJ, Floyd S, Machingura A, Mudziwapasi N, Gregson S, Glynn JR (2008). From affected to infected? Orphanhood and HIV risk among female adolescents in urban Zimbabwe. AIDS.

[24] Cornell M, McIntyre J, Myer L (2011). Men and antiretroviral therapy in Africa: our blind spot. Trop Med Int Health.

[25] Buzdugan R, McCoy SI, Watadzaushe C (2015). Evaluating the impact of Zimbabwe's prevention of mother-to-child HIV transmission program: population-level estimates of HIV-free infant survival pre-option A. PLoS One.

[26] Wagner AD, Mugo C, Njuguna IN (2016). Implementation and operational research: active referral of children of HIV-positive adults reveals high prevalence of undiagnosed HIV. J Acquir Immune Defic Syndr.

[27] Ferrand RA, Munaiwa L, Matsekete J (2010). Undiagnosed HIV infection among adolescents seeking primary health care in Zimbabwe. Clin Infect Dis.

[28] Ministry of Health and Child Care Zimbabwe (February, 2017). Operational and service delivery manual for the prevention, care and treatment of HIV in Zimbabwe. https://www.differentiatedcare.org/Portals/0/adam/Content/m2an155byU6RIoHeF4e4FQ/File/MSF%20Zim%20OSDM%20web%20revised.pdf.

[29] Njau B, Covin C, Lisasi E (2019). A systematic review of qualitative evidence on factors enabling and deterring uptake of HIV self-testing in Africa. BMC Public Health.

[30] Smith JA, Sharma M, Levin C (2015). Cost-effectiveness of community-based strategies to strengthen the continuum of HIV care in rural South Africa: a health economic modelling analysis. Lancet HIV.

[31] Tabana H, Nkonki L, Hongoro C (2015). A cost-effectiveness analysis of a home-based HIV counselling and testing intervention versus the standard (facility based) HIV Testing strategy in rural South Africa. PLoS One.

[32] Maheswaran H, Clarke A, MacPherson P (2018). Cost-effectiveness of community-based human immunodeficiency virus self-testing in Blantyre, Malawi. Clin Infect Dis.

