# Supplementary material for: Comparison of index-linked HIV testing for children and adolescents in health facility and community settings in Zimbabwe: findings from the interventional B-GAP study
Source: Lancet HIV. 2020 Nov 13;8(3):e138–48. doi: 10.1016/S2352-3018(20)30267-8 (PMC8011056; doi:10.1016/S2352-3018(20)30267-8)
Supplement: Supplementary appendix [file mmc1.pdf]

THE LANCET
HIV

Supplementary appendix

This appendix formed part of the original submission and has been peer reviewed. We post it as supplied by the authors.

Supplement to: Chikwari CD, Simms V, Kranzer K, et al. Comparison of index-linked HIV testing for children and adolescents in health facility and community settings in Zimbabwe: findings from the interventional B-GAP study. *Lancet HIV* 2020; published online Nov 13. https://doi.org/10.1016/S2352-3018(20)30267-8.

Table S1: Factors associated with choosing community testing by a lay worker vs facility testing at screening

Characteristic		Chose community testing by a lay worker<br>n =1487 children	Univariate analysis		Multivariate analysis<br>N = 4962	
		**n (%)**	**aOR (95% CI)**	**p value**	**aOR (95% CI)**	**p value**
**Index level variables**						
**Age (years)**	**0-18**	29 (2·0)	1·00 (ref)	-		
	**19-34**	383 (25·8)	1·18 (0·50-2·78)	0·71		
	**35-59**	945 (64·2)	1·65 (0·71-3·84)	0·24		
	**60+**	121 (8·1)	2·12 (0·83-5·42)	0·11		
**Sex**	**Male**	325 (21·9)	1·00 (ref)	-		
	**Female**	1162 (78·1)	0·91 (0·71-1·17)	0·48		
**Site**	**Rural**	401 (27·0)	1·00 (ref)	-	1·00 (ref)	-
	**Urban**	1086 973·0)	2·28 (1·78-2·91	<0·0001	2·10 (1·63-2·69)	<0·0001
**Highest level of education**	**None**	33 (2·2)	1·00 (ref)	-		
	**Primary**	468 (31·5)	0·64 (0·29-1·42)	0·27		
	**Secondary**	931 (62·6)	0·97 (0·44-2·11)	0·92		
	**Tertiary**	55 (3·7)	1·43 (0·57-3·61)	0·45		
**Mode of transport to facility[a]**	**By foot**	829 (55·8)	1·00 (ref)	-		
	**Public transport**	571 (38·4)	2·42 (1·92-3·04)	<0·0001		
	**By car**	11 (0·7)	0·71 (0·26-1·93)	0·50		
	**Other**	76 (5·1)	1·14 (0·68-1·93)	0·62		
**Cost to travel to facility (US$)**	**0**	915 (61·5)	1·00 (ref)	-	1·00 (ref)	-
	**>0**	572 (38·5)	1·24 (1·17-1·31)	<0·0001	1·21 (1·14-1·28)	<0·0001
**Duration since HIV diagnosis[b]**	**<1 year**	113 (7·8)	1·00 (ref)	-	1·00	
	**1-5 years**	678 (46·7)	1·49 (1·02-2·17)	0·040	1·59 (1·08-2·36)	0·020
	**≥6 years**	660 (45·5)	1·42 (0·97-2·08)	0·072	1·64 (1·10-2·44)	0·015
**Child level variables**						
**Sex[c]**	**Male**	751 (50·5)	1·00 (ref)	-		
	**Female**	735 (49·5)	0·91 (0·80-1·04)	0·16		
**Age[c]**	**2-5 years**	391 (26·3)	1·00 (ref)	-	1·00 (ref)	-
	**6-9 years**	396 (26·7)	1·27 (108-1·50)	0·0033	1·24 (1·04-1·47)	0·014
	**10-15 years**	532 (35·8)	1·40 (1·19-1·64)	<0·0001	1·39 (1·16-1·65)	0·0003
	**16-18 years**	167 (11·2)	1·27 (1·00-1·60)	0·04	1·19 (0·93-1·52)	0·17

**HIV status**[c]	**Known HIV negative >6 months**	854 (57·5)	1·00 (ref)	-	1·00 (ref)	-
	**Unknown**	632 (42·5)	1·31 (1·16-1·48)	0·0021	1·35 (1·19-1·63)	0·0018
**Relationship to index**[c]	**Nonbiological child**	588 (39·6)	1·00 (ref)	-		
	**Biological child**	898 (60·4)	1·09 (0·89-1·32)	0·41		
**Orphanhood status**[c]	**Not orphaned**	1134 (76·3)	1·00 (ref)	-		
	**Paternal Orphan**	248 (16·7)	1·01 (0·81-1·27)	0·93		
	**Maternal Orphan**	42 (2·8)	0·88 (·0·54-1·44)	0·60		
	**Double orphan**	62 (4·2)	1·48 (0·97-2·25)	0·70		
**Mother's HIV status**[d]	**HIV positive**	785 (64·1)	1·00 (ref)	-		
	**HIV Negative**	212 (17·3)	0·88 (0·67-1·15)	0·33		
	**Index doesn't know**	227 (18·6)	1·24 (0·94-1·64)	0·13		
**Any PMTCT history**[dce]	**Yes**	264 (30·9)	1·00 (ref)	-		
	**No**	527 (61·6)	1·34 (1·09-1·64)	0·0048		
	**Index doesn't know**	63 (7·4)	2·38 (1·49-3·81)	0·0003		
**Previous offer for HIV testing**[cdf]	**Yes**	456 (53·3)	1·00 (ref)	-		
	**No**	362 (42·3)	1·53 (1·23-1·90)	0·0001		
	**Index doesn't know**	36 (4·2)	1·27 (0·71-2·27)	0·42		

[a] *Only cost to travel to facility included in multivariate analysis due to collinearity with mode of transport to facility*
[b] *Missing data for 36 children* [ca] *Missing data for 1 child*
[c] *This question was only asked if the child was the biological child of the index no data for 632/1487*
[d] *Question only introduced March 1st therefore no data for 263/1487 children*
[e] *Excluded in multivariate model due to high number of missing data*
[f] *Only HIV status included in multivariate analysis due to collinearity with previous offer for HIV testing*

Table S2: Factors associated with choosing caregiver testing vs facility testing at screening

Characteristic		Chose caregiver testing n =300	Univariate analysis		Multivariate analysis N=3767	
		n (%)	aOR (95% CI)	p-value	aOR (95% CI)	p-value
**Index variables**						
**Age**	**0-18**	0 (0·0)	(empty)	-		
	**19-34**	97 (32·3)	1·00 (ref)	-		
	**35-59**	192 (64·0)	1·11 (0·72-1·71)	0·63		
	**60+**	11 (3·7)	0·65 (0·19-2·19)	0·48		
**Sex**	**Male**	38 (12·7)	1·00 (ref)	-	1·00 (ref)	-
	**Female**	262 (87·3)	1·76 (1·02-3·05)	0·043	1·92 (1·12-3·31)	0·018
**Site**	**Rural**	47 (15·7)	1·00 (ref)	-	1·00	-
	**Urban**	253 (84·3)	4·53 (2·55-8·06)	<0·0001	3·31 (1·75-6·23)	0·0002
**Highest level of education**	**None**	0	(empty)	-	(empty)	-
	**Primary**	70 (23·3)	1·00 (ref)	-	1·00 (ref)	-
	**Secondary**	212 (70·7)	2·29 (1·38-3·81)	0·0014	1·23 (0·73-2·06)	0·44
	**Tertiary**	18 (6·0)	4·88 (1·80-13·20)	0·0018	2·60 (0·82-8·17)	0·10
**Mode of transport to facility** [a]	**By foot**	174 (58·0)	1·00 (ref)	-		
	**Public transport**	120 (40·0)	2·42 (1·60-3·66)	<0·0001		
	**By car**	1 (0·3)	0·31 (0·04-2·32)	0·25		
	**Other**	5 (1·7)	0·35 (0·07-1·88)	0·23		
**Cost to travel to facility ($)** [a]	**$0·00**	175 (58·3)	1·00 (ref)	-	1·00 (ref)	-
	**>$0·00**	125 (41·7)	1·28 (1·16-1·42)	<0·0001	1·20 (1·08-1·33)	0·0005
**Duration since HIV diagnosis** [b]	**<1 year**	19 (6·3)	1·00 (ref)	-		
	**1-5 years**	137 (45·7)	1·79 (0·71-4·53)	0·22		
	**≥6 years**	118 (39·3)	1·84 (0·73-4·68)	0·20		
**Child variables**						
**Sex**	**Male**	133 (44·3)	1·00 (ref)	-		
	**Female**	167 (55·7)	1·17 (0·91-1·51)	0·23		
**Age**	**2-5 years**	81 (27·0)	1·00 (ref)	-		
	**6-9 years**	78 (26·0)	1·21 (0·89-1·64)	0·22		
	**10-15 years**	113 (37·7)	1·44 (1·05-1·96)	0·022		
	**16-18 years**	28 (9·3)	1·03 (0·62-1·70)	0·91		

**HIV status**	**Unknown**	174 (58·0)	1·34 (1·06-1·70)	0·016	1·73 (1·23-2·40)	0·0014
	**Known negative > 6 months**	126 (42·0)	1·00 (ref)	-	1·00 (ref)	-
**Relationship to index**	**Nonbiological child**	84 (28·0)	1·00	-	1·00 (ref)	-
	**Biological child**	216 (72·0)	1·83 (1·22-2·73)	0·0032	1·28 (0·86-1·90)	0·22
**Orphanhood status**	**Not orphaned**	253 (84·3)	1·00 (ref)	-		
	**Paternal Orphan**	40 (13·3)	0·73 (0·48-1·12)	0·15		
	**Maternal Orphan**	3 (1·0)	0·28 (0·09-0·88)	0·03		
	**Double orphan**	4 (1·3)	0·43 (0·16-1·17)	0·10		
**Mothers HIV status** [c]	**HIV positive**	200 (74·1)	1·00 (ref)	-		
	**HIV Negative**	35(13·0)	0·57 (0·31-1·05)	0·072		
	**Index doesn't know**	35 (13·0)	0·75 (0·45-1·27)	0·28		
**Any PMTCT History** [de]	**No**	119 (55·1)	1·00 (0·71-1·40)	1·00		
	**Yes**	80 (37·0)	1·00 (ref)	-		
	**Index doesn't know**	12 (7·9)	2·12 (1·04-4·33)	0·039		
**Previous offer for HIV testing** [df]	**No**	81 (37·5)	1·29 (0·89-1·86)	0·18		
	**Yes**	121 (56·0)	1·00 (ref)	-		
	**Index can't recall**	14 (6·5)	1·86 (0·83-4·17)	0·13		

[a] *Only cost to travel to facility included in multivariate analysis due to collinearity with mode of transport to facility*
[b] *Missing data for 26 children*
[c] *Missing data for 30/300 children as question was introduced into the study after 1/03/18*
[d] *This question was only asked if the child was the biological child of the index. No data for 89/300 children*
[e] *Excluded in multivariate model due to high number of missing data*
[f] *This question was only asked if the child was the biological child of the index (N=2739). No data for 84/300 children*
